# Prognostic significance of autophagy related genes in estrogen receptor positive tamoxifen treated breast cancer

**DOI:** 10.6026/97320630016710

**Published:** 2020-09-30

**Authors:** Alok Mishra, Ankit Pateriya, Anand Kumar Mishra, Ashutosh Shrivastava

**Affiliations:** 1 Center for Advance Research, Faculty of Medicine, King George's Medical University, Lucknow, Uttar Pradesh, India, 226003; 2 Department of Endocrine Surgery, Faculty of Medicine, King George's Medical University, Lucknow, Uttar Pradesh, India, 226003

**Keywords:** Breast Cancer, Estrogen Receptor, Tamoxifen resistance, Autophagy, Autophagy related genes, Kaplan-Meier Plot

## Abstract

Resistance to Tamoxifen constitutes a major therapeutic challenge in treating hormone sensitive breast cancer. The induction of autophagy has been shown to be involved as one of the mechanism responsible for Tamoxifen resistance. Autophagy related gene (ATG) members are the regulators and effectors of Macroautophagy process in the cellular systems. In this study, we evaluated the prognostic significance of ATGs in Tamoxifen treated breast cancer. The "Kaplan- Meier plotter" database was utilized to analyze the relevance and significance of ATGs mRNA expression to Relapse Free Survival in breast cancer patients. We used the data of patients who are Estrogen receptor positive and are treated with Tamoxifen. Hazard ratio and log-rank p-value were calculated using KM survival plots for various ATGs. Overexpressed ATG3, ATG 5, ATG 8B and PIK3R4 resulted in a poor prognosis. A gene signature of these ATGs predicts deteriorated RFS (p-value=8.3e-05 and HR=1.84 (1.35-2.51) and Distant Metastasis Free Survival (p value = 0.0027 and HR=2.03 (1.27-3.26). We report the distinct prognostic values of ATGs in patients of breast cancer treated with Tamoxifen. Thus, better understandings of the induction of autophagy pathway may potentially form the basis for use of autophagy inhibitors in the Tamoxifen treated breast cancer.

## Background

Breast cancer is one of the most commonly occurring cancers and according to estimation by the American Cancer Society, it is the second leading cause of cancer related mortality among American women [1]. According to an estimation in the United States, there were 268,600 new cases of invasive breast cancer resulting in approximately 42,000 deaths in the year 2019 [2]. There have been tremendous advancements in managing breast cancer involving early detection, advance therapeutic regimens, and a greater understanding of the molecular level details of its heterogeneity and biology. However, treating breast cancer that has become resistant to drugs or has metastasized remains a big challenge [3].

Estrogen signaling is critical in the pathogenesis of breast cancer, as ∼75% of breast cancers are positive for Estrogen Receptors (ER) [4]. These patients are generally treated by antiestrogens and Tamoxifen, a first line drug that is extensively used to treat ER positive breast cancer [5]. The Tamoxifen shares the structural similarity with estrogen and competes for available ER; thus modulates the ER function and result in reduced estrogen dependent cancer growth signaling [6]. Despite numerous breast cancer patients are benefited by Tamoxifen approximately 1/3rd of the patients acquire resistance to it and therefore a clinical challenge [7].

There are multiple mechanisms proposed for the development of Tamoxifen resistance. These mainly involve, Altered expression of ER, endocrine adaptation, Pharmacological tolerance, alteration in co-regulatory proteins, increased peptide growth signaling through EGFR/HER2, Insulin-like growth factor pathway and PIK3 pathways, etc [7]. In addition to these cell signaling, several cell functions such as disabled apoptosis and increased autophagy can contribute to acquired drug resistance in breast cancer [8]. More recently, the onset of autophagy has been considered a novel mechanism of drug resistance in various cancers [9].

The term autophagy usually indicates Macroautophagy, a type of autophagic process mediated by a unique assembly called the autophagosome. Autophagy is described as a self degradative process that takes place in a highly regulated manner [10]. Increasing evidence suggests that activation of autophagy in hormone responsive breast cancer cells can lead to the development of Tamoxifen resistance and result in the major therapeutic challenge [11-13]; Autophagy-related (ATG) proteins are key to the highly regulated execution of the autophagy. The autophagy-related family of proteins (ATG) constitute refined molecular machinery necessary for autophagosome formation, autophagosome-vesicle fusion, degradation, and nutrient recycling [14].

The onset and extent of autophagy critically determine the outcome of anticancer therapy. Given the central role of ATGs, we evaluated the prognostic significance of ATGs in the patient cohort of ER positive patients who have been treated with Tamoxifen therapy in this manuscript.

## Methodology

In this study, we used an online "Kaplan Meier Plotter" database to investigate the prognostic value and significance of mRNA expression of various ATGs to Relapse free survival (RFS). The estimation of RFS is critically important to determine the efficacy of new treatment. The KM-Plot database was constituted using Affymetrix chip-based gene expression data and survival information of a total of 3455 breast cancer patients [15]. This information for these patients in the KM plotter database extracted from the Gene Expression Omnibus (GEO, http://www.ncbi.nlm.nih.gov/geo/), Cancer Biomedical Informatics Grid (caBIG, https://biospecimens.cancer.gov/relatedinitiatives/overview/caBig.asp), and The Cancer Genome Atlas (TCGA, http://cancergenome.nih.gov) Breast cancer gene expression data resources [15]. Briefly, to determine the prognostic value of various ATGs, we primarily analyzed data from ER-positive patients who have received Tamoxifen and entered into the database (https://kmplot.com/analysis/index) and KM survival plots were extracted in which the numbers at the risk were mentioned below each plot diagram. Median gene expression values through default cut off in KM plotter separates patients in the high expression and low expression group for a particular gene. Hazard ratios (HR) and Log-rank p-values were calculated and ≤0.05 were considered statistically significant.

## Results:

In this study, we selected a cohort of patients that were ER-positive and treated with Tamoxifen. Depending upon the high or low expression of given ATG, RFS of patients was affected and in some cases resulted in the significantly increased HR. There are a total of at least 41 ATG genes have been identified [16]. Based on the executor mechanism of autophagy, there are several ATG core proteins based functional groups: (1) The ATG1/ Unc-51 like autophagy activating kinase (ULK) complex consisted of ULK1 or ULK2, ATG13, RB1CC1/FIP200, and ATG101; (2) the class III phosphatidylinositol 3-kinase (PIK3) complex consisted of BECN1/Beclin 1, ATG14, PIK3C3/VPS34, and PIK3R4/p150/VPS15); (3) the ATG12 conjugation system consisted of ATG7, ATG10, ATG12, ATG16L1, and ATG5; (4) the microtubule-associated protein 1 light chain 3 (LC3) conjugation system consisted of ATG4, ATG7, ATG3, WIPI2, and LC3 protein family; and (5) the ATG9 trafficking system consisted of ATG2A and ATG2B, WIPI4, and the transmembrane protein ATG9A [16,17].

**Figure 1:**
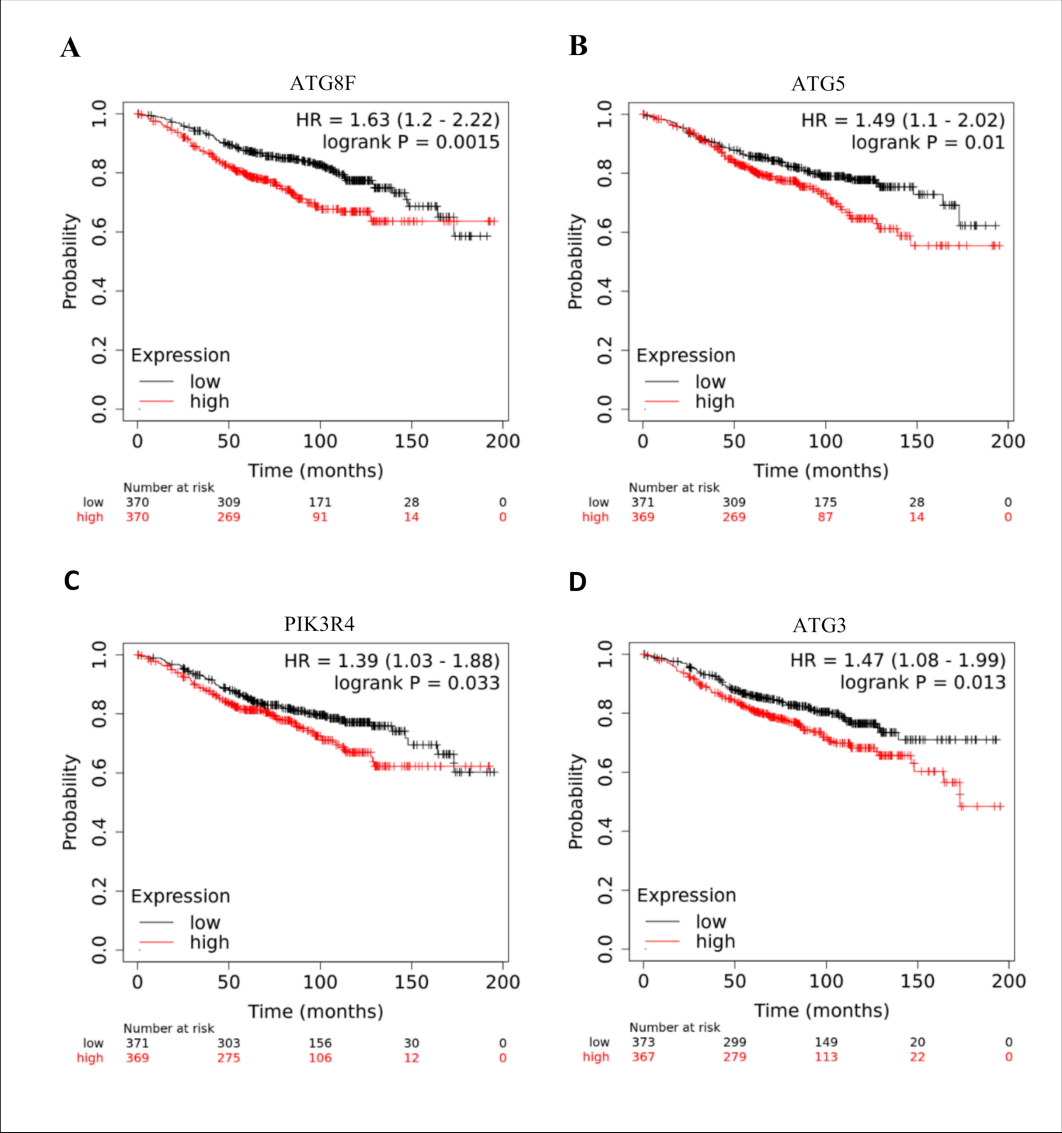
Graphical depiction of Prognostic significance of members of ATG family in predicting clinical outcome: Predictive value of ATG8F (A), ATG5 (B), PIK3R4 (C) and ATG3 (D) were assessed in ER+ve patients treated with Tamoxifen and followed over a period of >15 years.

We started by examining the prognostic value of various ATGs expression in the KM plot server (www.kmplot.com). Survival plots were extracted and values were noted (Table 1 - see PDF). The genes that had a significant p-values were looked in more detail (Figure 1). ATG8 and its mammalian homologs such as LC3, Gamma-aminobutyric acid receptor-associated protein (GABARAP) and Gamma-aminobutyric acid receptor-associated protein-like 2 (GABARAPL2) are ubiquitin like proteins and are involved in autophagosome formation [18]. Whenever autophagy is induced, the major regulatory pathways converge on the covalent lipidation of LC3 [18]. KM analysis revealed that ATG8F upregulation is related in tamoxifen treated breast cancer patients with a worsen prognosis, having a p-value=0.0015 HR=1.63(1.2-2.22) (Figure 1A).

ATG5 is considered as an essential molecule for the initiation of autophagy as it is mainly involved in the elongation of the autophagosome membrane [19]. ATG5 is the part of the ATG5-ATG12-ATG16 complex. This complex serves as a ubiquitin-like conjugation system that contributes to autophagic structures [20]. According to KM Plot analysis, high expression of ATG5 significantly decreases the median survival of Tamoxifen treated breast cancer patients with p-value=0.01 and HR=1.49 (1.1-2.02) (Figure 1B).

Autophagic process relies on the intricate interplay between membrane associated protein complexes. Phosphoinositide 3-kinase regulatory subunit4 (PIK3R4) is a member of Class 3 PIK complex, which produces Phosphatidylinositol 3-phosphate (PI3P) in the PAS or ER; and functions in the endocytic pathways. Autophagy process is initiated by ULK1 protein kinase complex and autophagy specific ClassIII Phosphatidylinositol 3-kinase complex (Ptdins3K-C1). PIK3R4 is a regulatory subunit of the PIK3 complex that mediates the formation of phosphatidylinositol 3-phosphate [16]. Its high expression resulted in poor prognosis of breast cancer patients treated with Tamoxifen as indicated by p-value=0.033 and HR =1.39 (1.39-1.88) (Figure 1C). ATG3 plays an important role in the process of autophagy by promoting the conversion between LC3I and LC3II during autophagy execution [21]. Thus ATG3 is a part of the LC3 conjugation system and it is essential for maintaining the mitochondrial integrity [21]. KM plot analysis revealed its elevated levels were related to poor prognosis of Tamoxifen treated breast cancer patients with p-value=0.013 and HR= 1.47 (1.08-1.99) (Figure 1D).

**Figure 2:**
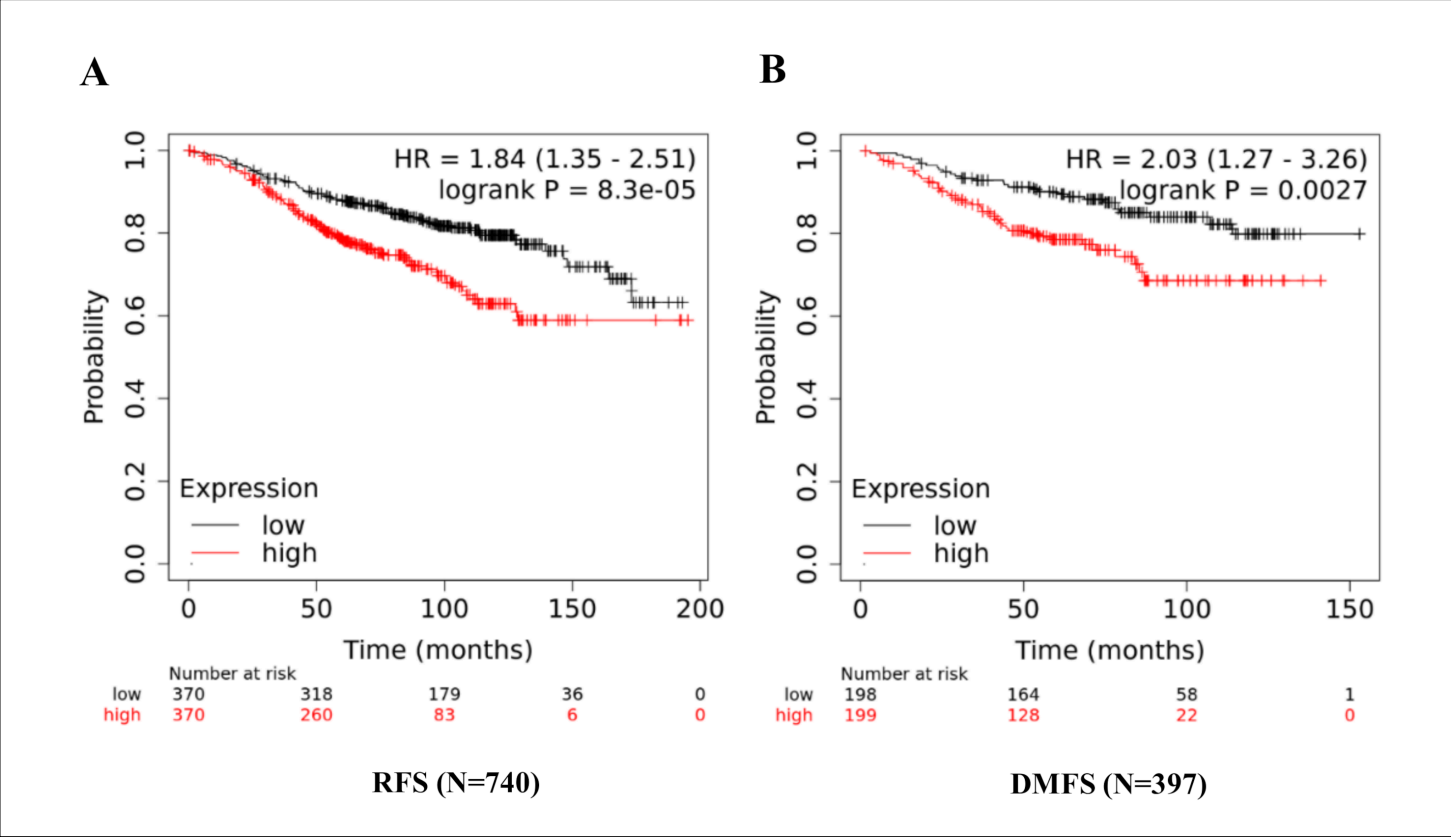
An ATG signature predicts poor clinical outcome in ER positive, Tamoxifen treated breast cancer patients. Combined signature designated as ATG signature of ATG8F+ATG5+PIK3R4+ATG3 significantly predicts the poor RFS (A) and poor DMFS (B).

After assessing the prognostic value of ATGs, as a next step; in order to increase the prognostic power of these individual markers out of these promising ones, we created an ATG based gene signature to predict prognosis of Tamoxifen treated breast cancer patients (Table 2 - see PDF). As indicated by the values: combined gene signature increased the statistical power of this signature with a highly significant p-value=8.3e-05 and HR=1.84 (1.35-2.51) (Figure 2A) to predict disease RFS or recurrence. ATG signature was found significantly predictive for distant metastasis free survival (DMFS) with a p-value= 0.0027 and HR= 2.03 (1.27-3.26) (Figure 2B). This ATG short signature may predict prognosis in ER positive breast cancer patients treated with anti estrogenic therapies.

**Figure 3:**
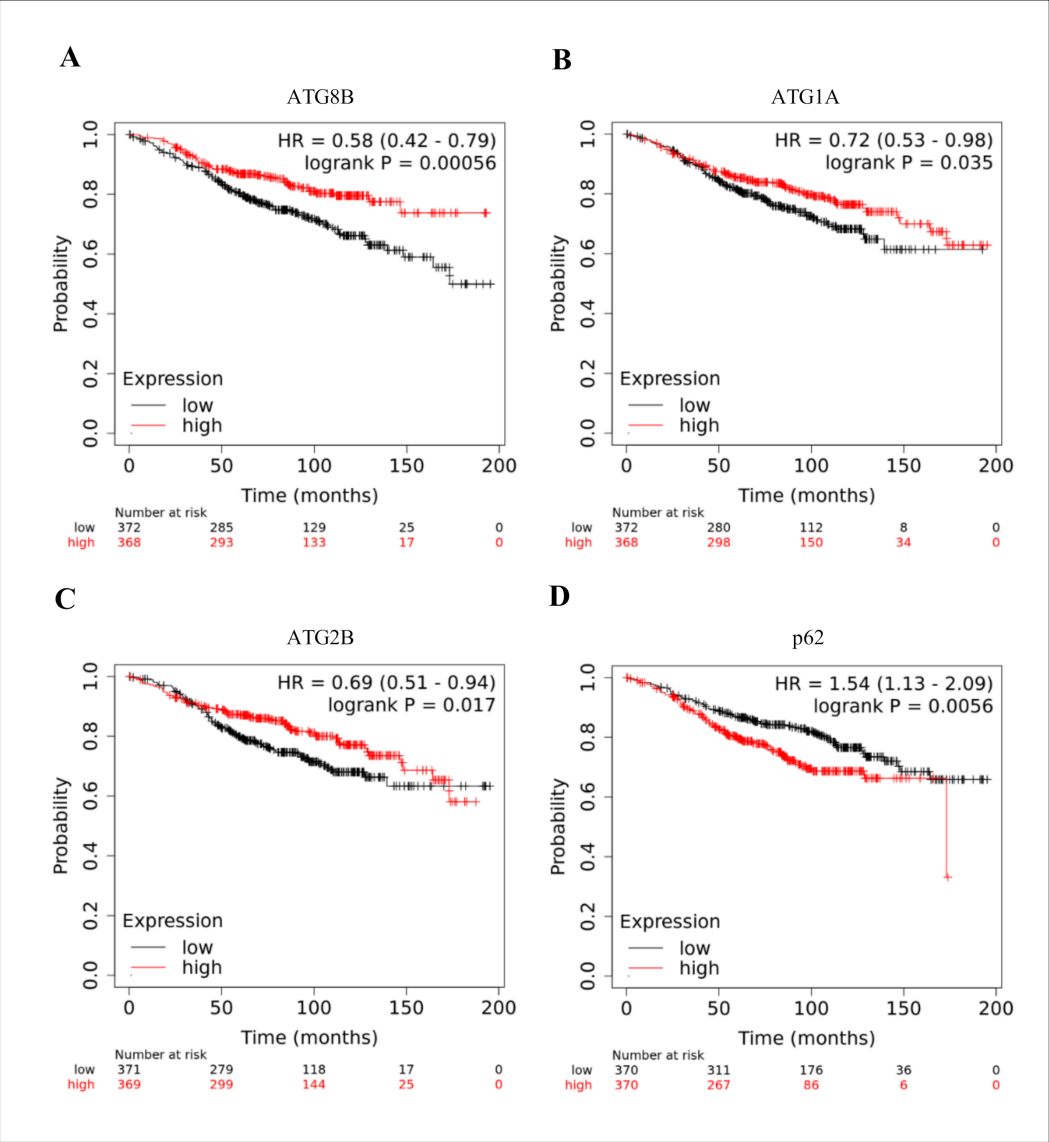
Graphical depiction of Prognostic values of members of ATG family and p62 in predicting clinical outcome: Predictive value of ATG8B (A), ATG1A (B), ATG2B (C) and p62 (D) was assessed in ER+ve patients treated with Tamoxifen and followed over a period of >15 years.

There were several ATGs like ATG8B, ATG1A and ATG2B where low expression level at transcriptomic levels resulted in poor prognosis of ER positive, Tamoxifen treated Breast cancer patients. ATG8B is the part of LC3 conjugation system [22]. ATG8B predominantly acts as an adapter for the autophagy machinery at the outer membrane of autophagosome, and is essential for autophagosome biogenesis/maturation and it also functions as an adaptor protein for selective Autophagy [22]. Overexpression of ATG8B resulted in poor prognosis of Tamoxifen resistance breast cancer patient, having p-value =0.00056 and HR= 0.58 (0.42-0.79) (Table 1 - see PDF) (Figure 3A).

ATG1A is a Serine/Threonine protein kinase and an integral part of the ULK-ATG13-ATG101-FIP200 complex. This complex is negatively regulated by mTORC1 in a nutrient dependent manner. This complex phosphorylates Beclin1. As shown in Table 1(see PDF), ATG1A/ULK1 have a p-value=0.035 and HR=0.72 (0.53-0.98) (Figure 3B). ATG2A and ATG2B proteins are essentially required for autophagy. These proteins also function in regulating the size and distribution of lipid droplets [23]. This protein is part of the ATG9/ATG12-WIPI complex, which is important for ATG9 recruitment to expand autophagosome, morphology and distribution [23]. ATG2B overexpression is correlated with poor RFS in Tamoxifen treated breast cancer patients, having p-value=0.017 and HR=0.69 (0.51-0.94) (Table 1 - see PDF) (Figure 3C).

In mammalian cells, autophagosome degradation can also be non-canonically driven by p62/sequestosome-1 (SQSTM1) [24]. Available literature suggests that p62 is an effector of autophagy and also a substrate [24]. p62 binds directly to ubiquitinated proteins and LC3, linking the ubiquitinated proteins to the autophagic machinery facilitate degradation of ubiquitinated protein aggregates by autophagy [25]. We found that p62 overexpression correlates with poor RFS having log-rank p-value=0.0056; HR=1.54 (1.53-2.09) (Figure 3D). This could translate into an important role of increased expression of p62 linking to Tamoxifen resistance in breast cancer.

## Discussion:

Breast cancer that has become treated to endocrine therapy poses an important clinical problem. The underlying mechanism of acquired or de-novo resistance to Tamoxifen is still poorly understood. It is important to note that if Autophagy is restored in treated breast cancer cells either by chemically inhibiting autophagy by Hydroxychloroquine or 3-Methyl Adenine [26] or through genetic restoration by RNAi interference of Beclin1. These interventions make resistant cells susceptible to apoptosis mediated cancer cell killing [27].

Since first discovered in 1970, Autophagy research has become a field of intense research. The investigation related to autophagy opened a new area of basic and translational research. It is now well established that autophagy play a significant role in normal physiology and disease, more importantly, it can modulate the outcome of anticancer therapy. Given the cytoprotective and cytodestructive roles of autophagy, this process controls the tumor growth and invasiveness, response of tumors to anticancer therapy, and resistance to it [28].

In this manuscript, we asked whether expression levels of ATGs predict the nature of autophagy. Also, can it be used to give us the information so we can predict the outcome of Tamoxifen therapy to resistant breast cancer? The KM plotter analysis points that significant upregulation of ATG3, ATG5, PIK3R4, and ATG8B to be linked with poor prognosis of Tamoxifen treated breast cancer patients. This kind of upregulation can directly result in autophagy upregulation as a survival mechanism in Tamoxifen treated Breast cancer cells. The activation of the canonical pathway of upregulation culminates in the increased lipidation of ATG8B. This kind of autophagy mediated cell survival can interfere with the efficacy of Tamoxifen and can result in decreased RFS and decreased DMFS as the KM plotter prediction shows (Figure 2 and Table 2 - see PDF). We conclude that active autophagy is involved in Tamoxifen resistant cells and results in the worsen outcome. The gene signature of combined mean expression data with P value= 8.3e-05 and HR=1.84 (1.35-2.51) (Table 2 - see PDF) may be useful in making treatment choices for managing drug resistant breast cancer.

We compared the data of ER positive breast cancer patient's cohort receiving Tamoxifen (n=751) to the patient cohort of ER positive patients not treated with Tamoxifen or any endocrine therapy (n=657). KM plot analysis revealed that only ATG8F upregulation correlate with poor prognosis in the cohort of ER positive Tamoxifen untreated patients (p-value=2.1e-0.6 HR=1.48 (1.26-1.75). This entails that involvement of autophagy regulation by multiltiple ATGs is specific to ER positive and Tamoxifen treated patients cohort only. Further, KM plot data take mean mRNA expression of a particular gene and that may not be reflective of its protein levels. Moreover ATG8F activity in a given system is dependent on its lipidation that is a post-translational modification [18].

We observed that low expression of ATG8B/GABARAPL1 to link with poor RFS. It has been shown that GABARAPL1 is a tumor suppressor and this function to be independent of autophagosome formation in hormone responsive breast cancer cells [29]. Moreover, the latest study shows that GABARAPs and LC3s play contrasting roles while regulating ULK1 for autophagy initiation [30]. Therefore, it can be concluded that hyperactivate canonical autophagy pathway play a crucial role in poor prognosis in Tamoxifen treated breast cancer. Furthermore; contrasting results in the case of ATG8B are inconsequential in the context of autophagy induction in Tamoxifen treated breast cancer cells.

Sequestosome1 (p62/SQSTM1) is a scaffold protein that is activated under stress conditions [24]. It has been shown that p62 can directly interact with LC3 for autophagosome formation and play a role in tumor development [31]. Other than LC3, the link of p62 overexpression of p62 and its association with poor prognosis of Tamoxifen resistant cells show the important role that p62 may play during Tamoxifen resistance.

Various studies have shown that increased autophagy accompanies Tamoxifen resistance in breast cancer [11,13,32]. Given the opposing role of autophagy in cancer, to elucidate the role of autophagy in cancer dependent context is critical. Based on the dual role of autophagy, there are autophagy activators that are promising targets in the clinic as well as autophagy inhibitors [26]. For example, mTOR belongs to the family of phosphatidylinositol-3-kinase-related kinases and coordinates eukaryotic cell growth and metabolism [33]. mTOR pathway deregulation can lead to cancer. Rapamycin is a known inhibitor of mTOR and has been tested clinically to treat cancer [26,33].On the other hand activating autophagy can induce cell death in target cancer cells and autophagy inhibitors have also been tried clinically [26].

There is need of more data from experiments on tissue samples from Tamoxifen resistance breast cancer samples and correlating autophagic structures, levels of ATGs relating to patient's prognosis, and survival conclusively. Retrospective studies could be planned to correlate ATGs expression with patients clinical information such as age, grading and staging of tumor resistant to tamoxifen therapy. ATG based signature can be of utility to make a case for using autophagy inhibitors to increase the susceptibility of Tamoxifen resistant cells to undergo active cell death and better prognosis.

## Conclusion:

The ongoing research on regulation of autophagy will likely provide new information to predict the response to therapeutic interventions. Autophagy based markers could be used as a companion to diagnostic platforms to faithfully predict the outcome to drug resistance to Tamoxifen and drug resistance in general. Nevertheless, in vivo and in vitro experiments and multi-center randomized controlled clinical trials are still needed preceding their use in clinical settings.

## Funding:

The Laboratory of Dr. Ashutosh Shrivastava is supported by King George's Medical University Intramural Grant Award and a research grant from CCRH, Ministry of AYUSH, Government of India (Z.28015/01/2018-HPC-EMR-AYUSD-D).

## Availability of data and materials:

Not applicable

## Authors' contributions:

Conceived and designed the study: A.S. and A.M.; Data analysis: A.S., A.M., A.P. and A.K.M. Wrote the manuscript: A.S., A.M. and A.P. All authors concur on conclusions of this study.

## Ethics approval and consent to participate:

Not applicable

## Patient consent for publication:

Not applicable
